# Serum osteopontin as a prognostic biomarker in acute exacerbations of chronic obstructive pulmonary disease

**DOI:** 10.3389/fimmu.2025.1708595

**Published:** 2025-11-11

**Authors:** Kai-Shu Ma, Li-Na Li, Yi-Cheng Ma, Ru-Liu Fan, Gang Chen, Hui Zhao, Kai-Xin Qu, Lin Fu

**Affiliations:** 1 Department of Respiratory and Critical Care Medicine, Funan County People’s Hospital, Fuyang, Anhui, China; 2 Department of Respiratory and Critical Care Medicine, The Second Affiliated Hospital of Anhui Medical University, Hefei, Anhui, China; 3 Institute of Respiratory Diseases, The Second Affiliated Hospital of Anhui Medical University, Hefei, Anhui, China; 4 Thoracic Surgery Department, The Second Affiliated Hospital of Anhui Medical University, Hefei, Anhui, China

**Keywords:** COPD, osteopontin, death, acute exacerbation, cohort study

## Abstract

Osteopontin, a phosphorylated glycoprotein, is highly expressed in lung tissues and is elevated in inflammatory diseases. However, its role in acute exacerbation of chronic obstructive pulmonary disease (AECOPD) remains unclear. A total of 281 AECOPD patients, 89 stable COPD (SCOPD) cases, and 89 healthy volunteers were enrolled in this prospective cohort according to the inclusion and exclusion criteria. Demographic information and clinical features were obtained from electronic medical records systems. Fasting venous blood was collected on the day of admission, and baseline serum osteopontin was measured using an enzyme-linked immunosorbent assay. The primary endpoints—death, frequency of acute exacerbations, and hospital length of stay—were evaluated through a follow-up study. Baseline serum osteopontin levels were higher in AECOPD patients compared to SCOPD patients and healthy volunteers. Linear regression analysis revealed positive associations between serum osteopontin and severity scores in AECOPD patients, and an inverse correlation of serum osteopontin and pulmonary function in SCOPD patients. In addition, baseline serum osteopontin levels were elevated in AECOPD patients with poorer prognosis. Logistic regression analysis indicated that serum osteopontin was positively correlated with the risks of death and acute exacerbations in the first year. Receiver operating characteristic (ROC) curve analysis suggested that the predictive ability of serum osteopontin for poor prognosis was comparable to that of the COPD Assessment Test (CAT) score and superior to the modified Medical Research Council (mMRC) score among AECOPD patients. Our findings indicate that baseline serum osteopontin is positively associated with severity scores and adverse clinical outcomes, highlighting its potential value as a surrogate prognostic biomarker in AECOPD patients.

## 1 Introduction

Chronic obstructive pulmonary disease (COPD) is a common chronic lung disease characterized by partially reversible airflow limitation, persistent inflammation, and abnormal respiratory symptoms (1, 2). The global prevalence of COPD is estimated at approximately 12.16% (3), with a slightly higher incidence in men than in women (4). At present, COPD ranks as the fourth most common chronic disease (5). Its high prevalence, associated disability, and mortality impose a substantial economic burden on both individuals and society (6). The pathological mechanism of COPD is complex; inhalation of harmful particles, gases, outdoor, and both outdoor and indoor air pollution can contribute to COPD through various mechanisms (7–9). Therefore, early disease detection and prognosis prediction are essential for identifying individuals at higher risk of progressing to an acute exacerbation of COPD (AECOPD).

Increasing studies have investigated potential biomarkers for estimating the prognosis in COPD patients. Currently, attention has been paid to osteopontin, which is a phosphorylated glycoprotein and highly expressed in the bone and many tissues (10). It is known that osteopontin is implicated in a number of physiopathological processes, including inflammation, bone homeostasis, cell survival and adhesion, and immune response (11, 12). Osteopontin plays important roles in many diseases, including rheumatoid arthritis, osteoarthritis, fibrosis, obesity, diabetes, neuroinflammation, and cancers (13–16). Further studies have also shown that osteopontin is expressed in various cell types, such as osteoblasts, fibroblasts, macrophages, neutrophils, lymphoid cells, and epithelial cells (17–19).

Osteopontin is also produced in lung tissues and is strongly correlated with various pulmonary diseases. Under physiological conditions, osteopontin is expressed at low levels in macrophages, bronchioles, fibroblasts, club cells, and endothelial cells of the lungs. On the contrary, cell stress and inflammation activation increase osteopontin expression (20–22). In pulmonary fibrosis, osteopontin expression is elevated in macrophages, fibroblasts, and epithelial cells (23, 24). Increased osteopontin contributes to pulmonary fibrosis through epithelial–mesenchymal transition, and osteopontin knockdown significantly alleviates bleomycin-evoked pulmonary fibrosis in mice (24). In addition, osteopontin is increased in the macrophages of patients with coronavirus disease 2019 (25). It has been observed that osteopontin expression is higher in senescent pulmonary vascular cells of mice with pulmonary hypertension than in young mice (26). Moreover, osteopontin levels in sputum supernatant are upregulated in COPD patients compared with healthy subjects (27). Animal experiments have also shown increased osteopontin expression in the lung tissues of COPD mice (28). These findings underscore the significant role of osteopontin in COPD.

However, the exact role of osteopontin in AECOPD remains unclear. No evidence has yet demonstrated a correlation between serum osteopontin and prognosis in AECOPD patients. In the current study, serum osteopontin concentrations were measured in AECOPD patients, healthy volunteers, and stable COPD (SCOPD) cases. The relationship between serum osteopontin and disease progression was also explored in AECOPD patients. Our results provide the first evidence that serum osteopontin expression may reflect disease severity in AECOPD.

## 2 Materials and methods

### 2.1 Subjects

AECOPD patients were selected from the Anhui COPD cohort (AHCC), which was described in previous studies from our laboratory (29, 30). AECOPD patients were recruited at the time of diagnosis in the Department of Respiratory Medicine from three tertiary hospitals in Anhui Province. The follow-up investigation was conducted 2 years later. All AECOPD patients had a confirmed COPD diagnosis consistent with the Global Initiative for Chronic Obstructive Lung Disease (GOLD) criteria (31). All included AECOPD patients, who had no other respiratory diseases, were over 18 years old and voluntarily participated in the follow-up study. On the day of admission, fasting venous blood samples were collected from AECOPD patients before treatment. In addition, to evaluate serum osteopontin expression, age- and sex-matched healthy participants and SCOPD patients were enrolled. The hospital stay following the acute exacerbation was recorded. Follow-up assessments were conducted every 3 months after the first exacerbation, during which hospitalization duration, exacerbations, and deaths were recorded. Patients who experienced two or more exacerbations per year were classified as having frequent exacerbations (32). Moreover, patients whose hospitalization lasted more than 13 days (third quartile) in the first year were categorized as prolonged hospital stays (33). Based on the inclusion and exclusion criteria, 350 AECOPD patients were initially selected from the Anhui COPD cohort. As shown in the flow diagram of recruitment and follow-up, 281 eligible AECOPD cases were ultimately included in the current clinical investigation (**Supplementary Figure S1**).

### 2.2 Data collection

On the first day of admission, demographic data were obtained using a questionnaire. All COPD patients and healthy volunteers underwent medical history and physical examination. Routine blood tests were performed, and biochemical indicators were collected, including liver function, renal function, and inflammatory cytokines. In addition, pulmonary function was assessed in SCOPD patients by a professional nurse in accordance with the American Thoracic Society guidelines (34). Forced expiratory volume in 1 s (FEV1) and forced vital capacity (FVC) were recorded. SCOPD patients were categorized into four groups based on GOLD grades: I (FEV1% ≥ 80%), II (50% ≤ FEV1% < 80%), III (30% ≤ FEV1% < 50%), and IV (FEV1% < 30%). On admission, the symptoms and severity of AECOPD patients were assessed using the COPD Assessment Test (CAT) score (35). Based on the CAT score, AECOPD patients were classified into four groups: low (CAT score < 10), medium (10 ≤ CAT score < 20), high (20 ≤ CAT score < 30), and very high (30 ≤ CAT score < 40). Moreover, the degree of breathlessness was evaluated using the modified Medical Research Council (mMRC) score (36). Based on mMRC scores, AECOPD patients were classified into five subgroups: 0 (breathlessness only during vigorous exercise); 1 (expiratory dyspnea when walking quickly on level ground or climbing a gentle slope); 2 (walking slower than peer or needing to stop when climbing a gentle slope); 3 (needing breath after walking 100 m or several minutes on level ground); 4 (severe breathlessness at rest, or when dressing or undressing).

### 2.3 Enzyme-linked immunosorbent assay

Fasting blood samples were collected from all participants at 6:00 in the morning. All samples were kept at room temperature for 2 h, then centrifuged at 3,500 rpm for 10 mins. The resulting serum samples were extracted and stored in − 80°C until analysis. Serum osteopontin levels were measured using a commercially available enzyme-linked immunosorbent assay (ELISA) kit (CSB-E08392h, CUSABIO, http://www.cusabio.cn/) following the manufacturer’s standardized protocols (37, 38). Briefly, serum samples were diluted and added to the assay plate. The biotin-conjugated antibody was added and incubated at 37°C for 1.5 h. After washing, HRP-avidin was added to each well and incubated for an additional 1.5 h. The TMB substrate was then added, and the reaction was stopped by adding the stop solution. Lastly, absorbance was measured at 450 nm using a microplate reader.

### 2.4 Statistics analysis

The normality of the data was assessed using the Kolmogorov–Smirnov test, with a *p*-value greater than 0.05 considered indicative of a normal distribution. Normally distributed data are presented as mean ± standard deviation and compared using one-way analysis of variance (ANOVA). Nonnormally distributed data are expressed as median and analyzed using the Wilcoxon rank-sum test. Categorical variables are presented as counts (percentages), and differences between groups were assessed using a nonparametric test. In addition, the relationships between baseline serum osteopontin expression and CAT, mRMC, and pulmonary function parameters were examined using Pearson correlation and linear regression analyses. To evaluate the robustness of the statistical models, stratified analyses were performed according to age, gender, and smoking status. Based on serum osteopontin tertiles, AECOPD patients were categorized into three groups: low, serum osteopontin ≤ 1.118 ng/mL; medium, serum osteopontin 1.118~1.579 ng/mL; and high, serum osteopontin ≥ 1.579 ng/mL. The association of serum osteopontin expression and poor prognosis was evaluated using logistic regression analysis. The predictive capacity for major in-hospital clinical outcomes was assessed with a receiver operating characteristic (ROC) curve. Predictive performance was expressed as the area under the curve (AUC). A *p*-value less than 0.05 was considered statistically significant.

## 3 Results

### 3.1 Clinical data and demographic features

In this investigation, 90 healthy volunteers, 89 SCOPD patients, and 281 AECOPD patients were enrolled. Ages were higher in SCOPD and AECOPD patients than in healthy volunteers (**Table 1**). Significant differences were observed among the three groups in men’s smoking status, hypertension, coronary heart disease, and cerebrovascular disease (**Table 1**). In addition, several clinical parameters—including ALT, AST, uric acid, urea nitrogen, creatinine, creatine kinase, creatine kinase isoenzyme, myoglobin, lactate dehydrogenase, and interleukin-6 (IL-6)—differed significantly among the groups (**Table 1**).

**Table 1:** Demographic characteristics of participators at baseline.

	Healthy volunteers	SCOPD	AECOPD	*P*
N	90	89	281	
Age, year	62.6±0.51	71.0±0.81	74.3±0.50	0.025
Male, n (%)	49 (53.3)	49 (55.1)	208 (72.0)	<0.001
Smoking status, n (%)				<0.001
Former	8 (8.7)	56 (62.9)	116 (41.3)	
Current	22 (23.9)	25 (28.1)	49 (17.4)	
None	60 (65.2)	8 (9.0)	116 (41.3)	
Comorbidities, n (%)				
Hypertension	17 (18.5)	43 (48.3)	124 (42.9)	<0.001
Coronary heart disease	1 (1.1)	16 (18.0)	35 (12.1)	<0.001
Diabetes mellitus	7 (7.6)	5 (5.6)	24 (8.3)	0.778
Cerebrovascular disease	2 (2.2)	10 (11.2)	30 (10.4)	0.021
Inhaled therapy, n (%)				
ICS+LABA		16 (18.0)	15 (5.2)	NA
LABA+LAMA		8 (9.0)	43 (14.9)	NA
ICS+LABA+LAMA		54 (60.7)	80 (27.7)	NA
Alanine aminotransferase (U/L)	25.7±0.24	23.3±0.22	31.0±0.73	<0.001
Aspartate aminotransferase (U/L)	25.7±0.15	27.3±0.18	33.3±0.36	<0.001
Uric acid (μmol/L)	321.0 (257.0, 365.00	353.0 (298.0, 410.5)	289.0 (233.0, 369.0)	<0.001
Urea nitrogen (mmol/L)	5.8 (5.0, 6.9)	6.0 (4.9, 7.3)	6.2 (4.7, 8.2)	<0.001
Creatinine (μmol/L)	60.0 (50.0, 73.0)	69.0 (63.5, 83.5)	66.0 (53.2, 82.0)	<0.001
Creatine kinase (U/L)	101.0 (67.0, 131.0)	92.0 (69.5, 128.5)	59.0 (39.0, 85.0)	<0.001
Creatine kinase isoenzyme (U/L)	16.0 (13.0, 19.0)	16.0 (12.5, 19.0)	14.0 (11.0, 18.0)	<0.001
Myoglobin (ng/mL)	24.2 (18.6, 31.0)	31.2 (24.1, 38.7)	36.4 (25.3, 52.4)	<0.001
Lactate dehydrogenase (U/L)	181.3±3.44	192.4±5.68	196.5±3.95	<0.001
IL-6 (pg/mL)	1.9 (1.1, 3.8)	3.6 (1.8, 6.1)	19.0 (4.3, 62.2)	<0.001
FEV1 (L)	2.7±0.06	1.6±0.07		NA
FVC (L)	3.5±0.09	2.8±0.09		NA
FEV1 (%)	112.3±1.89	60.8±2.72		NA
FEV1/FVC (%)	79.2±0.56	54.0±1.13		NA

ALT, alanine aminotransferase; AST, aspartate aminotransferase; ICS, Inhaled corticosteroids; LABA, long-acting beta agonist; LAMA, long-acting muscarinic antagonist; IL-6, Interleukin-6.

### 3.2 Serum osteopontin levels in SCOPD and AECOPD patients with varying disease severity

**Figure 1:**
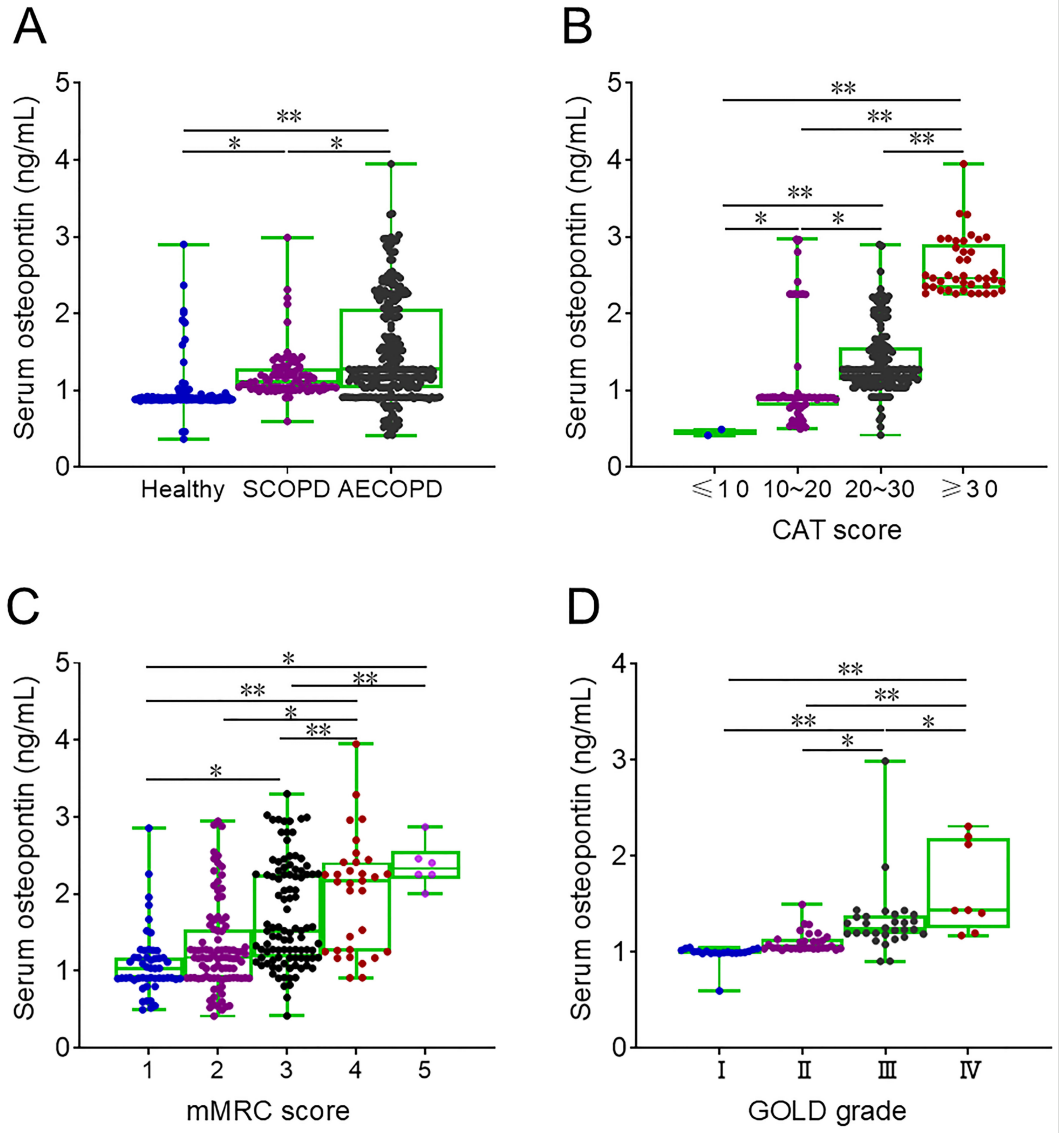
Baseline serum osteopontin expression in AECOPD patients. **(A–D)** Serum samples were collected, and baseline serum osteopontin expression was determined by ELISA. Differences in serum osteopontin levels were then compared. **(A)** Baseline serum osteopontin expression in healthy volunteers, SCOPD patients, and AECOPD patients. **(B)** Baseline serum osteopontin expression in AECOPD patients with distinct CAT scores. **(C)** Baseline serum osteopontin expression in AECOPD patients with distinct mMRC scores. **(D)** Baseline serum osteopontin expression in SCOPD patients with distinct GOLD grades. ^*^p < 0.05; ^**^p < 0.01.

Serum osteopontin expression was elevated in patients with SCOPD and COPD compared to healthy volunteers and was highest in AECOPD patients (**Figure 1A**). In AECOPD patients, serum osteopontin levels were compared across different severity scores and were found to increase progressively with higher CAT and mMRC scores (**Figures 1B, C**). Moreover, in SCOPD patients, serum osteopontin levels were assessed according to GOLD stages, showing that higher GOLD stages were associated with higher serum osteopontin levels (**Figure 1D**).

### 3.3 Association between serum osteopontin expression on the day of admission and severity

**Figure 2:**
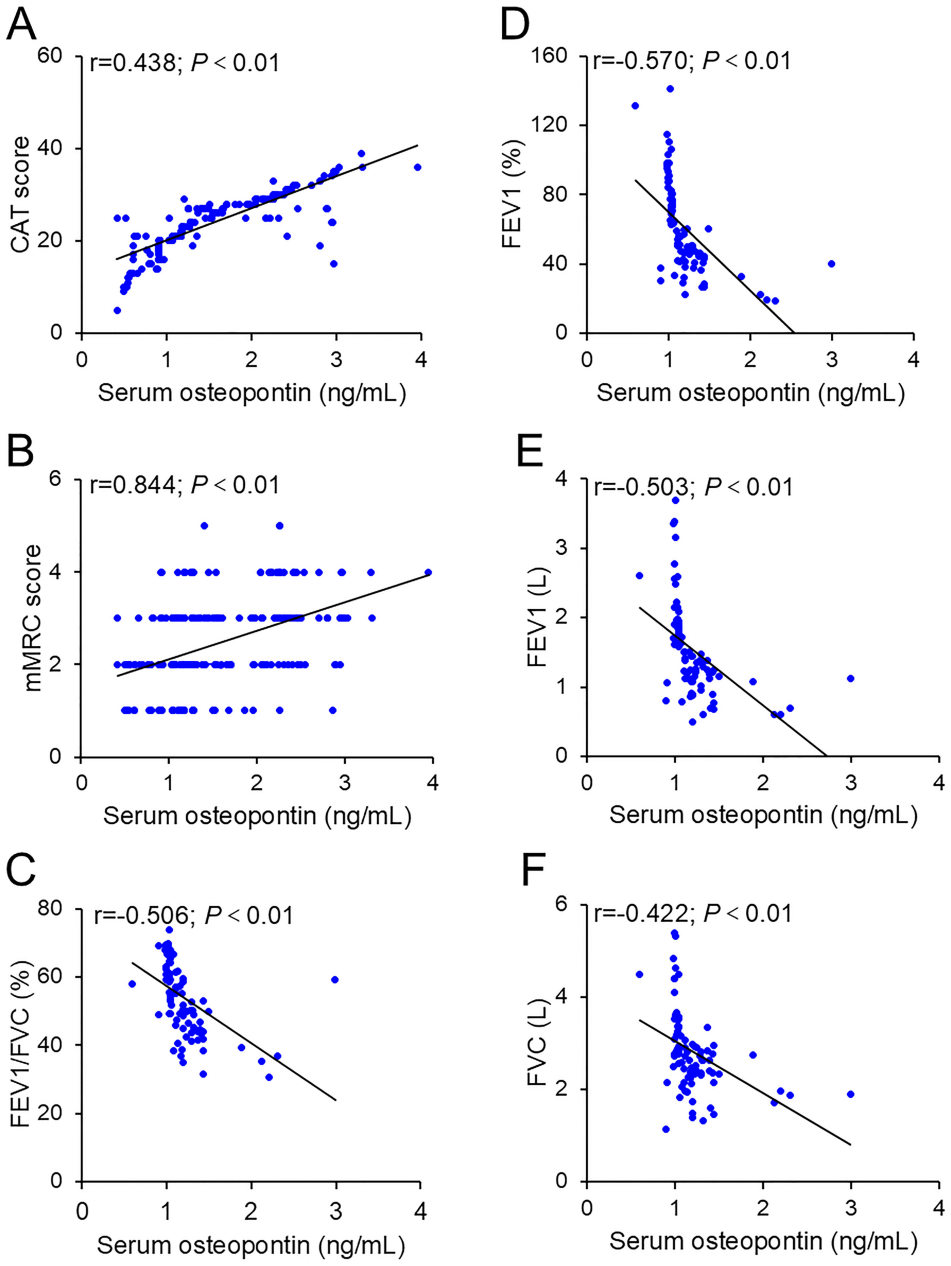
Correlation between baseline serum osteopontin expression and severity. **(A, B)** Correlations between serum osteopontin and CAT scores **(A)** or mMRC scores **(B)** were estimated using Pearson correlation analysis. **(C–F)** Associations between serum osteopontin and pulmonary function parameters were also evaluated using Pearson correlation analysis, including FEV1/FVC% **(C)**, FEV1% **(D)**, FEV1 **(E)**, and FVC **(F)**.

The correlations between serum osteopontin concentration on the day of admission and severity scores were evaluated by Pearson correlation analysis among AECOPD patients. As shown in **Figures 2A, B**, serum osteopontin expression was positively associated with CAT (*r* = 0.438; *p <* 0.01) and mMRC (*r* = 0.844; *p <* 0.01) scores among AECOPD patients. In addition, Pearson correlation analysis confirmed inverse relationships between serum osteopontin expression on the day of admission and FEV1/FVC% (*r* = − 0.506; *p <* 0.01), FEV1% (*r* = − 0.570; *p <* 0.01), FEV1 (*r* = − 0.503; *p <* 0.01), and FVC (*r* = − 0.422; *p <* 0.01) in SCOPD patients (**Figures 2C–F**). The correlations of serum osteopontin expression with severity scores and pulmonary function indicators were further evaluated using linear regression analysis. Univariate linear regression analysis revealed positive correlations between serum osteopontin expression and CAT (*β* = 6.948; 95% CI: 6.426–7.469) as well as mMRC (*β* = 0.617; 95% CI: 0.469–0.764) in AECOPD patients (**Table 2**). Moreover, each 1 ng/mL increase in baseline serum osteopontin was significantly associated with reductions of 1.052, 1.118, 45.305, and 16.758 in FEV1, FVC, FEV1%, and FEV1/FVC%, respectively, in SCOPD patients (**Table 2**). Several confounding variables, such as age, sex, smoking status, comorbidities, and inhaled therapy, were adjusted. Multivariate linear regression further validated the abovementioned relationships (**Table 2**). To further evaluate the relationship between serum osteopontin and severity, stratified analysis was conducted in different subgroups. The results indicated that serum osteopontin remained positively associated with the severity scores of AECOPD cases (**Supplementary Table S1**) and inversely correlated with pulmonary function parameters in COPD patients (**Supplementary Table S2**). Age, sex, and smoking status had a weak influence on the association of serum osteopontin and severity.

**Table 2.** Associations between serum osteopontin and severity scores.

	Variables		Estimated changes by serum osteopontia, β (95% CI)	*P*
AECOPD	Osteopontin	N	281	
	Unadjusted	CAT	**6.948 (6.426, 7.469)**	**＜0.001**
		mMRC	**0.617 (0.469, 0.764)**	**＜0.001**
	Adjusted	CAT	**6.958 (6.404, 7.512)**	**＜0.001**
		mMRC	**0.643 (0.487, 0.798)**	**＜0.001**
SCOPD	Osteopontin	N	89	
	Unadjusted	FEV1	**-1.052 (-1.437, -0.667)**	**＜0.001**
		FVC	**-1.118 (-1.629, -0.606)**	**＜0.001**
		FEV1%	**-45.305 (-59.216, -31.394)**	**＜0.001**
		FEV1/FVC%	**-16.758 (-22.839, -10.677)**	**＜0.001**
	Adjusted	FEV1	**-1.195 (-1.584, -0.806)**	**＜0.001**
		FVC	**-1.371 (-1.870, -0.872)**	**＜0.001**
		FEV1%	**-44.646 (-59.148, -30.144)**	**＜0.001**
		FEV1/FVC%	**-15.741 (-21.945, -9.537)**	**＜0.001**

* Adjusted for age, sex, smoking status, comorbidities, and inhaled therapy.

Data in bold denote statistically significant results.

### 3.4 Serum expressions of osteopontin in AECOPD patients with different prognoses

**Figure 3:**
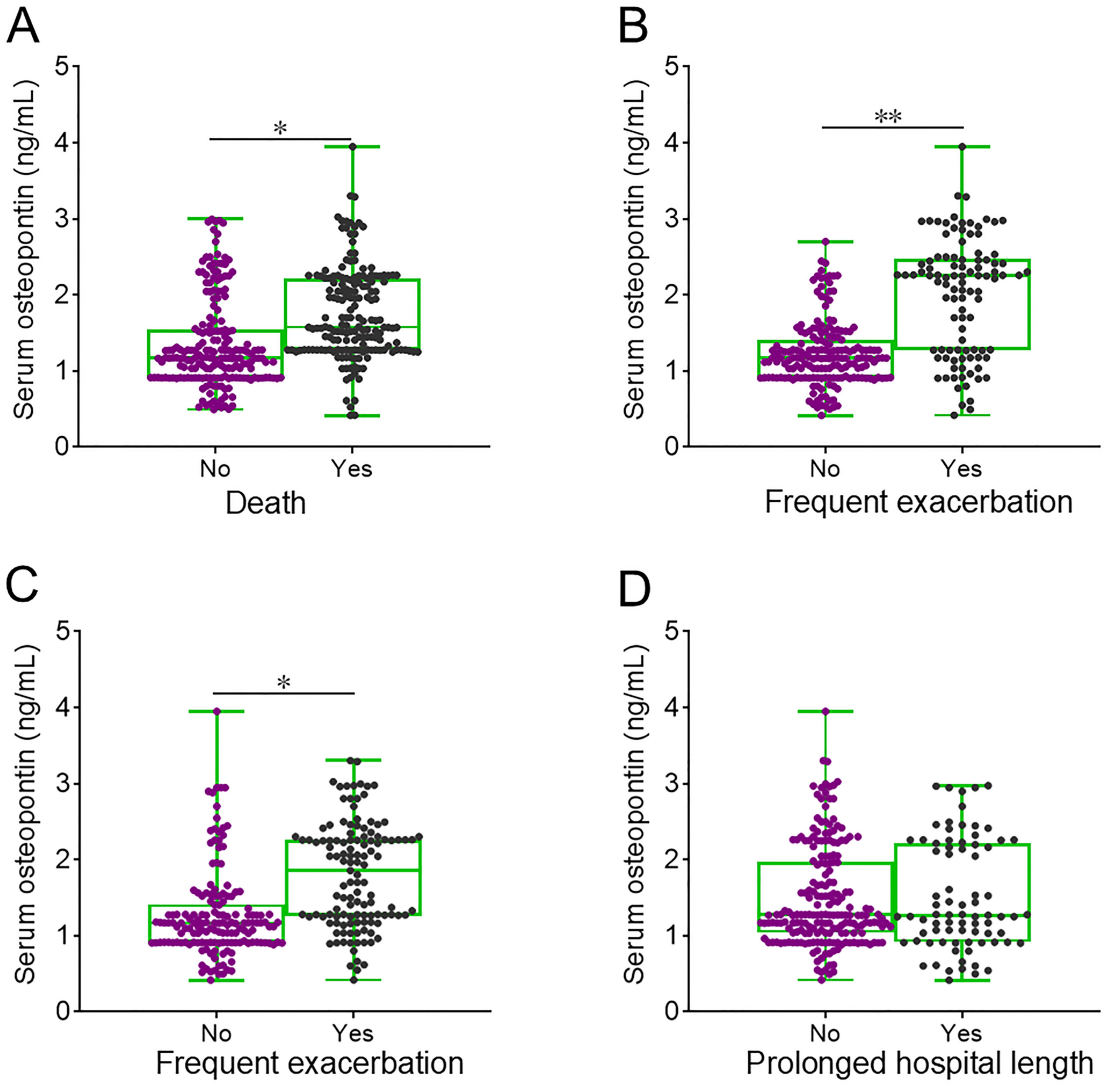
Baseline serum osteopontin expression in AECOPD patients with different clinical outcomes. **(A–D)** Serum osteopontin expression was compared on the day of admission among AECOPD patients who experienced different clinical outcomes: death **(A)**, frequent exacerbation in the first year **(B)**, frequent exacerbation in the second year **(C)**, and prolonged hospital stay **(D)**. ^*^p < 0.05; ^**^p < 0.01.

The level of serum osteopontin on the day of admission was elevated in deceased AECOPD patients compared to those who survived (**Figure 3A**). The baseline serum osteopontin expression was then compared between AECOPD patients with and without frequent exacerbations. The data revealed that serum osteopontin levels on the day of admission were higher in AECOPD patients who experienced frequent exacerbations compared to those with infrequent exacerbations in both the first and second years (**Figures 3B, C**). Moreover, no significant difference in baseline serum osteopontin was observed between AECOPD patients with and without prolonged hospital stays (**Figure 3D**).

### 3.5 Correlation between serum osteopontin expression on the day of admission and prognosis

The number of cases who experienced death at the follow-up stage was 13 (13.8%), 26 (27.7%), and 45 (48.4%) in the low, medium, and high groups, respectively (**Table 3**). Compared to the low group, univariate logistic regression analysis indicated that the relative risk of death was increased in medium (RR = 2.382; 95% CI: 1.137–4.992) and high (RR = 5.841; 95% CI: 2.863–11.917) groups (**Table 3**). Additionally, the numbers of acute exacerbation in the first year were 42 (44.7%), 55 (58.5%), and 88 (94.6%), while the cases of acute exacerbation in the second year were 43 (45.7%), 63 (67.0%), and 76 (81.7%) in the low, medium, and high groups, respectively (**Table 3**). Univariate logistic regression analysis showed that serum osteopontin concentration on the day of admission was positively correlated with the risk of acute exacerbations in the first and second years among AECOPD cases. After controlling for several confounding variables, multivariate logistic regression analysis still confirmed that high serum osteopontin concentration increased the risk of death and acute exacerbations in the first year of AECOPD patients (**Table 3**). However, no significant association was found between serum osteopontin expression and prolonged hospital stay in AECOPD patients (**Table 3**).

**Table 3.** Associations between serum osteopontin and prognostic outcomes in AECOPD patients at follow-up stage.

Variables	Serum osteopontin			*P*trend
	Low	Medium	High	
N	93	94	94	
Death				
N, (%)	13 (13.8)	26 (27.7)	45 (48.4)	**<0.001**
Unadjusted RR	Ref (1.0)	**2.382 (1.137, 4.992)**	**5.841 (2.863, 11.917)**	**<0.001**
Adjusted RR	Ref (1.0)	1.820 (0.715, 4.635)	**4.048 (1.125, 14.569)**	**0.025**
Acute exacerbation in the first year				
N, (%)	42 (44.7)	55 (58.5)	88 (94.6)	**<0.001**
Unadjusted RR	Ref (1.0)	1.746 (0.980, 3.111)	**21.790 (8.108, 58.560)**	**<0.001**
Adjusted RR	Ref (1.0)	1.481 (0.660, 3.324)	**16.608 (3.909, 70.556)**	**<0.001**
Acute exacerbation in the second year				
N, (%)	43 (45.7)	63 (67.0)	76 (81.7)	**<0.001**
Unadjusted RR	Ref (1.0)	1.713 (0.949, 3.095)	**3.769 (1.940, 7.324)**	**<0.001**
Adjusted RR	Ref (1.0)	1.360 (0.625, 2.961)	2.666 (0.834, 8.526)	0.086
Prolonged hospital length				
N, (%)	27 (28.7)	22 (23.4)	28 (30.1)	0.573
Unadjusted RR	Ref (1.0)	0.757 (0.393, 1.458)	1.053 (0.561, 1.977)	0.870
Adjusted RR	Ref (1.0)	0.744 (0.319, 1.736)	0.952 (0.290, 3.125)	0.974

* Adjusted for age, sex, smoking status, comorbidities, and inhaled therapy.

Data in bold denote statistically significant results.

### 3.6 Predictive value of serum osteopontin expression for adverse prognosis in AECOPD patients

**Figure 4:**
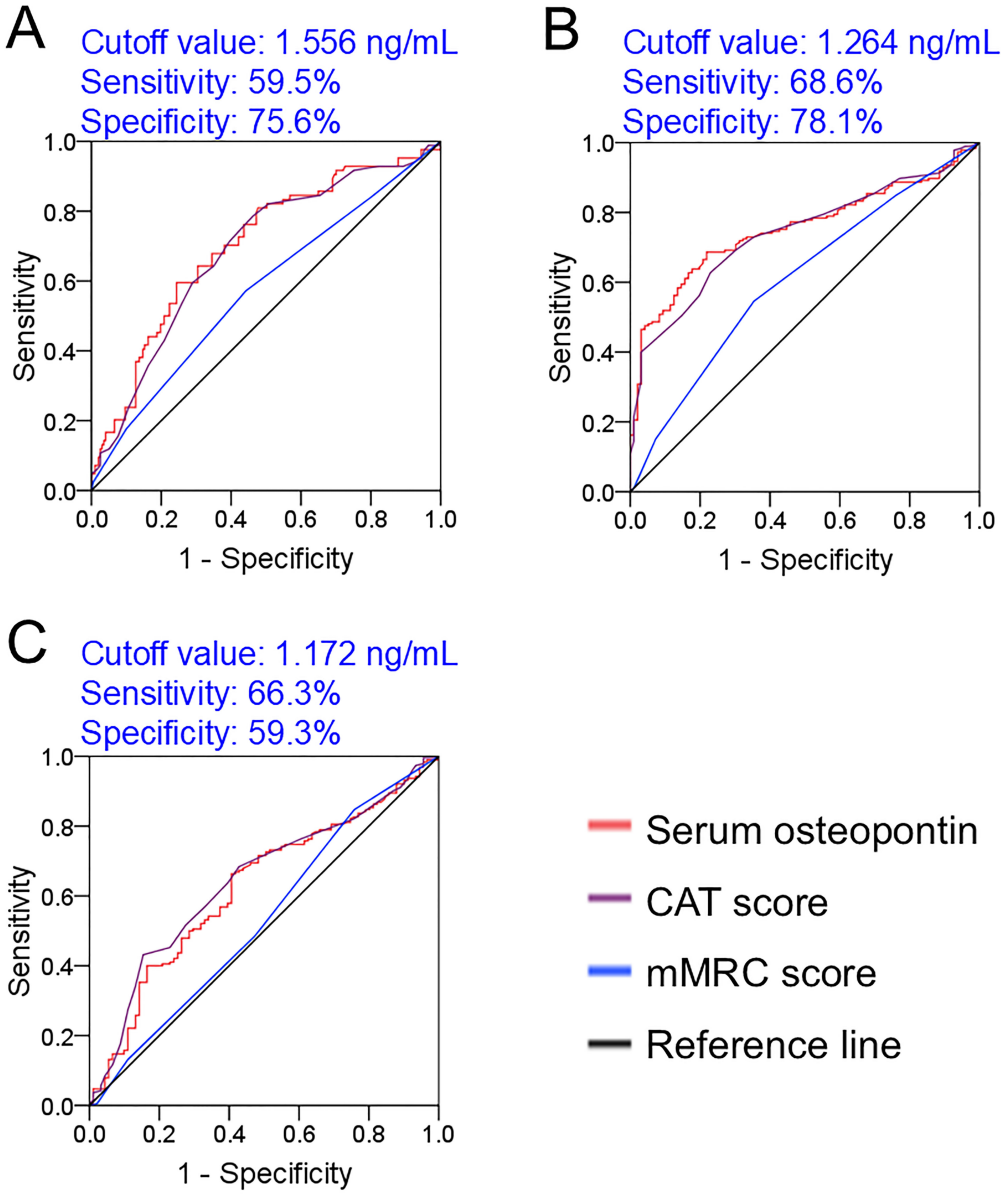
Predictive power of baseline serum osteopontin expression for poor prognosis in AECOPD patients. **(A–C)** ROC curves were used to estimate the predictive capacity of baseline serum osteopontin for adverse clinical outcomes. **(A)** Prediction of death. **(B)** Frequent exacerbation in the first year. **(C)** Frequent exacerbation in the second year.

The predictive values of serum osteopontin concentration and severity scores for in-hospital adverse prognosis were analyzed via ROC among AECOPD patients. As shown in **Figure 4A**, the AUCs for death were as follows: serum osteopontin, 0.701; CAT score, 0.687; and mMRC score, 0.574, indicating that the predictive power of serum osteopontin and CAT score for death was higher compared with the mMRC score within 2 years among AECOPD patients. Moreover, the predictive power of serum osteopontin concentration (AUC = 0.752) for acute exacerbation in the first year was comparable to that of the CAT score (AUC = 0.739), and higher than that of the mMRC score (AUC = 0.608) (**Figure 4B**). Additionally, there was no significant difference in AUC for acute exacerbation in the second year between serum osteopontin expression (AUC = 0.624) and the CAT score (AUC = 0.644), while the predictive capacity was reduced in the mMRC score (AUC = 0.531) among AECOPD patients (**Figure 4C**).

## 4 Discussion

This study primarily measured serum osteopontin levels on the day of admission and analyzed their relationship with disease severity and prognosis in AECOPD patients. Our findings revealed, for the first time, that upregulated serum osteopontin expression is associated with increased clinical severity and adverse outcomes in AECOPD patients over a 2-year period.

Osteopontin, a phosphorylated glycoprotein, is highly expressed in lung tissue and is present in various cell types, including fibroblasts, macrophages, neutrophils, lymphocytes, and epithelial cells (10, 17–19). Numerous studies have demonstrated that osteopontin plays key roles in the pathophysiology of pulmonary diseases. Under normal conditions, osteopontin is expressed in most cells (20–22). Pulmonary osteopontin expression is increased in both the mouse model and patients with lung adenocarcinoma (39). Moreover, pulmonary osteopontin levels are upregulated in macrophages and epithelial cells of patients with fibrosis (23, 24). In sepsis-incurred acute lung injury, both osteopontin mRNA and protein levels are elevated in mouse lungs (40). Pulmonary osteopontin expression is also increased in patients with COPD (41). These findings suggest a vital role for osteopontin in respiratory diseases. However, the precise relationships between serum osteopontin expression, disease severity, and prognosis have rarely been investigated in AECOPD cases. Our current projects found that serum osteopontin expression on the day of admission was elevated in AECOPD patients compared to SCOPD patients and healthy volunteers. Among AECOPD patients, serum osteopontin levels were higher in those with elevated CAT and mMRC scores. Correlative analysis revealed that serum osteopontin expression was inversely associated with pulmonary function in SCOPD patients and positively correlated with CAT and mMRC scores in AECOPD patients. These findings confirm that serum osteopontin expression on the day of admission is positively related to clinical severity in AECOPD patients.

Increasing evidence suggests that osteopontin expression is associated with the prognosis of various diseases. A recent study reported that osteopontin expression in macrophages is positively correlated with cancer stage and tumor grade in patients with head and neck squamous cell carcinoma (42). Overexpression of the osteopontin gene has been linked to shorter overall survival and progression-free interval (43). In addition, elevated plasma osteopontin levels are associated with higher mortality in sepsis patients (44). Based on these findings, we speculated that serum osteopontin expression on the day of admission may be associated with an increased risk of poor outcomes in AECOPD patients. We found that serum osteopontin on the day of admission was elevated in deceased cases and in patients with frequent exacerbations. Logistic regression analysis further confirmed the positive association of baseline serum osteopontin expression with both mortality and the number of exacerbations. ROC curve analysis showed that the predictive power of serum osteopontin for poor prognosis was similar to that of the CAT score and higher than that of the mMRC score. These data indicate that serum osteopontin expression on the day of admission is positively correlated with in-hospital poor prognosis in AECOPD patients.

Our study demonstrated that serum osteopontin levels were positively correlated with disease severity and poor prognosis in AECOPD patients. However, the precise mechanism by which osteopontin influences prognosis in these patients remains unclear. Previous studies have shown that osteopontin can activate nuclear factor-κ B (NF-κB), a key transcription factor that regulates downstream target genes, including proinflammatory cytokines and chemokines, thereby triggering inflammatory cascades and promoting COPD progression (45). Dysregulated inflammation is recognized as a central mechanism in the initiation and progression of COPD (46). Inflammatory cytokines not only activate immune responses but also alter the levels of eosinophils, lymphocytes, and neutrophils in the human body. An abnormal inflammatory state can result in alveolar damage and airway stenosis, as well as trigger acute exacerbations and worsen prognosis in COPD patients (47–49). In addition, several studies have confirmed that osteopontin is a major component of the senescence-associated secretory phenotype (SASP), can promote tissue remodeling, and regulate fibroblast function (26, 50). Emerging evidence highlights the pivotal role of cellular senescence in the progression of COPD (51). Senescent cells exacerbate inflammation, promote malignant transformation, alter the local microenvironment, and impair pulmonary structure and function (52). Thus, these findings suggest that elevated osteopontin may contribute to adverse COPD progression, at least in part through mechanisms involving inflammation and cellular senescence.

Although this investigation provides new insights into the role of osteopontin in COPD progression, several limitations should be acknowledged. First, this was a single-center study; more AECOPD patients should be recruited from multiple hospitals. Second, as a clinical epidemiological study, it only explored the association between serum osteopontin and AECOPD, and the precise mechanism underlying serum osteopontin upregulation remains unclear. Only animal and cellular experiments can reveal the underlying molecular mechanisms. Third, this study analyzed only the correlation between serum osteopontin and AECOPD patients. In reality, many other inflammatory cytokines and components of the SASP are simultaneously elevated in AECOPD cases. The potential confounding effects of these factors on the relationship between serum osteopontin and prognosis cannot be fully excluded. Epidemiological research can generally estimate only one or a few factors at a time. Therefore, to some extent, a new mixed forecasting model may help address this issue.

Clinically, the diagnosis of AECOPD primarily relies on medical history and clinical manifestations. Acute exacerbations are the main cause of death and progressive decline in pulmonary function. By improving early detection and risk assessment in AECOPD patients, clinicians can better identify those at risk of poor clinical outcomes (53). However, the similarities between AECOPD and other pulmonary diseases make diagnosis challenging. In addition, in the stable and early stages, COPD symptoms are often mild and can be easily overlooked by both patients and clinicians. Thus, earlier diagnosis, timely assessment, and therapeutic interventions can help improve prognosis and reduce mortality (54). Clinical examinations, pulmonary function tests, and severity score evaluations all play important roles in identifying the risk of acute exacerbations. However, the predictive power of these assessments may be limited by complex indicators and equipment constraints. Single, readily available biomarkers can help compensate for these limitations. Serum osteopontin is easily and inexpensively measured, with diagnostic performance comparable to the CAT score and superior to the mMRC score. Therefore, measuring serum osteopontin may provide a valuable tool for enhancing diagnostic accuracy and predicting poor prognosis in AECOPD patients.

## 5 Conclusion

In summary, by comparing the baseline expression of serum osteopontin in AECOPD, SCOPD, and healthy volunteers, we found that serum osteopontin is significantly upregulated in AECOPD and SCOPD patients compared to healthy volunteers, particularly in AECOPD patients. Additionally, baseline serum osteopontin expression is positively correlated with disease severity and may serve as a prognostic biomarker for poor clinical outcomes in AECOPD patients during hospitalization. These findings highlight the potential clinical utility of serum osteopontin in diagnosis and prognosis assessment for AECOPD patients. Nevertheless, further studies are needed to explore the association of these findings with patient phenotypes and the underlying mechanisms driving osteopontin elevation.

## Data Availability

Publicly available datasets were analyzed in this study. The raw data supporting the conclusions of this article will be made available by the authors, without undue reservation.
