# Supplementary material for: Serum osteopontin as a prognostic biomarker in acute exacerbations of chronic obstructive pulmonary disease
Source: Front Immunol. 2025 Nov 11;16:1708595. doi: 10.3389/fimmu.2025.1708595 (PMC12643881; doi:10.3389/fimmu.2025.1708595)
Supplement: Supplementary file 1 [file Table1.doc]

Supplemental Table 1. Stratified analyses for the associations between serum osteopontin and severity scores in AECOPD patients.

| Stratification characteristic | | CAT | mMRC |
| --- | --- | --- | --- |
| Age (years) |  |  |  |
|  | ≤ 74.0 | **7.037 (6.254, 7.810)** | **0.736 (0.514, 0.957)** |
|  | ＞74.0 | **6.853 (6.042, 7.665)** | **0.589 (0.366, 0.813)** |
|  | *P*_interaction_ | 0.132 | 0.098 |
| Gender |  |  |  |
|  | Male | **6.952 (6.351, 7.552)** | **0.641 (0.462, 0.819)** |
|  | Famale | **6.485 (4.914, 8.057)** | **0.739 (0.327, 1.150)** |
|  | *P*_interaction_ | **0.010** | **0.032** |
| Smoking status |  |  |  |
|  | None | **6.688 (5.679, 7.696)** | **0.699 (0.434, 0.964)** |
|  | Former | **7.401 (6.649, 8.154)** | **0.631 (0.382, 0.880)** |
|  | Current | **6.440 (4.747, 8.133)** | **0.602 (0.200, 1.004)** |
|  | *P*_interaction_ | 0.201 | 0.325 |
