# Supplementary material for: Serum osteopontin as a prognostic biomarker in acute exacerbations of chronic obstructive pulmonary disease
Source: Front Immunol. 2025 Nov 11;16:1708595. doi: 10.3389/fimmu.2025.1708595 (PMC12643881; doi:10.3389/fimmu.2025.1708595)
Supplement: Supplementary file 2 [file Table2.doc]

Supplemental Table 2. Stratified analyses for the association between serum osteopontin and pulmonary function in SCOPD patients.

| Stratification characteristic | | FVC (L) | FEV1 (L) | FEV1/FVC (%) | FEV1 (%) |
| --- | --- | --- | --- | --- | --- |
| Age (years) |  |  |  |  |  |
|  | ≤ 71.0 | **-1.156 (-1.784, -0.528)** | **-0.975 (-1.471, -0.479)** | **-12.213 (-19.838, -4.588)** | **-35.327 (-51.264, -19.390)** |
|  | ＞71.0 | **-2.090 (-3.357, -0.823)** | **-2.303 (-3.036, -1.571)** | **-41.714 (-55.698, -27.729)** | **-99.432 (-131.570, -67.295)** |
|  | *P*_interaction_ | **＜0.001** | 0.054 | **0.001** | **0.023** |
| Gender |  |  |  |  |  |
|  | Male | -3.878 (-6.592, -1.078) | -3.403 (-12.039, -1.568) | **-51.083 (-112.365, 2.546)** | **-9.616 (-42.365, -1.326)** |
|  | Famale | **-1.324 (-1.830, -0.817)** | **-1.168 (-1.563, -0.772)** | **-15.656 (-22.000, -9.311)** | **-42.874 (-57.116, -28.631)** |
|  | *P*_interaction_ | **0.020** | **0.046** | 0.058 | 0.145 |
| Smoking status |  |  |  |  |  |
|  | None | 0.172 (-31.628, 31.971) | 0.066 (-30.096, 30.228) | 5.634 (-582.756, 594.024) | -44.340 (-196.168, 107.488) |
|  | Former | **-1.186 (-1.781, -0.591)** | **-0.937 (-1.392, -0.481)** | **-9.581 (-16.893, -2.268)** | **-35.616 (-52.639, -18.593)** |
|  | Current | **-1.609 (-2.971, -0.247)** | **-1.657 (-2.671, -0.642)** | **-30.894 (-45.073, -16.715)** | **-53.012 (-88.083, -17.941)** |
|  | *P*_interaction_ | **0.042** | 0.085 | **0.005** | **0.020** |
