# Supplementary figures and images for: Serum osteopontin as a prognostic biomarker in acute exacerbations of chronic obstructive pulmonary disease
Source: Front Immunol. 2025 Nov 11;16:1708595. doi: 10.3389/fimmu.2025.1708595 (PMC12643881; doi:10.3389/fimmu.2025.1708595)

**Supplemental Figure**


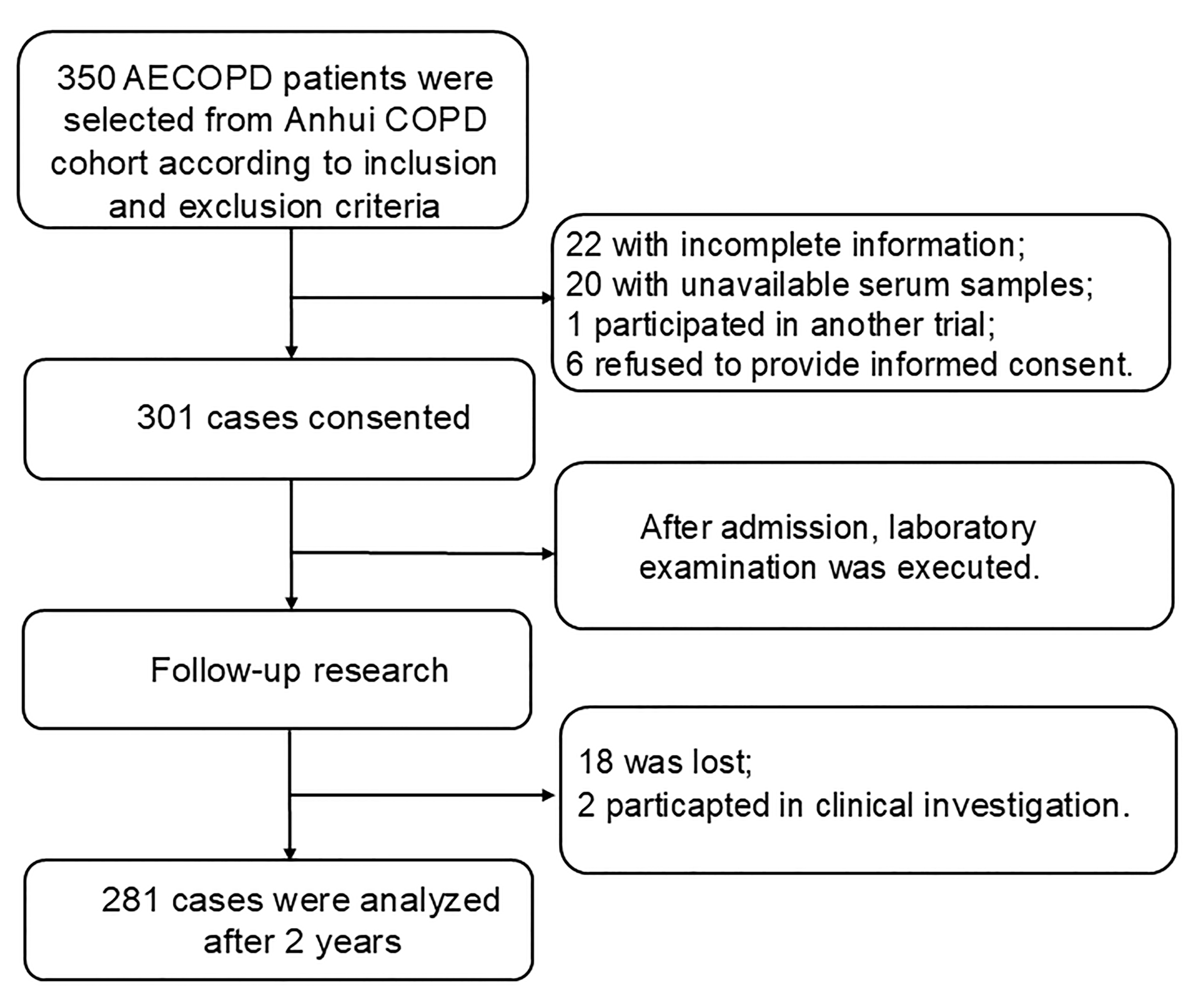


**Supplemental Figure 1. Flow diagram of recruitment and follow-up research.**

Supplement: Supplementary file 3 [file Supplementaryfile1.doc]
